# Dental Association

**Published:** 1862-12

**Authors:** 


### DENTAL ASSOCIATIONS.

Quite an active movement is being made in Iowa for the formation of a State Dental Association, one that we doubt not will effect the desired object. We are much pleased to see this effort on behalf of our western brethren. They have the ability, energy and men to form good associations all over the West. Indiana has a State association, St. Louis has her local society, Iowa is taking up the same work, and we trust ere long Illinois will report herself in the same line of duty.

No one knows the power, efficiency and value of associated effort till he tests it for himself. By that means only can men know their own ability, and by that means will they become most rapidly developed in professional attainments. We hope our brethren every where will stir up to this work of associated effort. T.

